# Determinants of early cessation of exclusive breastfeeding practices among rural mothers from Jaffna District of Sri Lanka

**DOI:** 10.1186/s13006-023-00575-z

**Published:** 2023-08-15

**Authors:** Kandeepan Karthigesu, Sandrasegarampillai Balakumar, Vasanthy Arasaratnam

**Affiliations:** Department of Biochemistry, Faculty of Medicine, University of Jaffna, Jaffna, Sri Lanka

**Keywords:** Exclusive breastfeeding, Breastfeeding duration, Infants

## Abstract

**Background:**

Sri Lanka is an upper-middle-income country with excellent health statistics. However, 2016 Demographic and Health Survey data have shown 82% and 64% of mothers exclusively breastfed infants aged 0–6 months and 4–5 months, respectively. The short duration of exclusive breastfeeding (EBF) has an impact on the growth and development of babies. Since no studies have been reported on EBF practices of the rural mothers in Jaffna District, an administrative district among 25 districts of Sri Lanka, this study aimed to assess the factors influencing the early cessation of EBF.

**Methods:**

For this community-based cross-sectional study, 338 mother–child pairs were selected from 2013–14. EBF was defined as children not receiving any food or drink, including complementary foods, formula milk or milk products except for medicines and vitamins or mineral drops, other than breast milk since birth. Socio-economic and demographic factors, the influence of the mode of delivery, and knowledge on EBF were obtained using an interviewer-administered questionnaire. The details of EBF and reasons for the cessation of breastfeeding before six months were obtained from a subgroup of mothers (*n* = 208). Multivariate analysis was performed to explore the correlates of breastfeeding.

**Results:**

In this study, 71.2% (95% CI 64.5, 77.2) had practiced EBF for six months. Early discontinuation of EBF was practiced by employed mothers (AOR 4.3; 95% CI 1.3, 13.9), mothers of low birth weight babies (AOR 3.6; 95% CI 1.6, 8.2) and those who experienced Cesarean section birth (AOR 2.9; 95% CI 1.2, 6.9). The EBF practiced by mothers of rural Jaffna was not associated with the gender of the babies, type of family, number of children in a family, religion of the household, knowledge on EBF, or family income.

**Conclusion:**

The prevalence of EBF up to six months was low in rural Jaffna, and it was influenced by employment, birthweight of the babies, and the mode of delivery. To enhance EBF, the Regional Directorate of Health Service, Jaffna, should take necessary action with policymakers to increase maternity leave for at least six months, reduce the Cesarean section rate, and provide nutritional support to pregnant mothers.

## Background

Breast milk is a ready food for infants that provides cost-effective nutrition important for children’s health and development [1]. The World Health Organization (WHO) defines exclusive breastfeeding (EBF), as a mother giving only breast milk, without any additional food or liquid, even water, with the exception of drops or syrups consisting of vitamins, minerals, supplements, oral rehydration solution or medicines [2]. Feeding breast milk provides essential nutrients for growing infants [1] with protective elements [3]. Globally, 60% of mothers did not breastfeed their babies in the first hour of life, and 41% of mothers failed to provide EBF for infants under six months of age [4].

According to the World Breastfeeding Trends Initiative report, Sri Lanka achieved first green nation status in advancing breastfeeding protection, promotion and support out of 97 countries [5]. It needs improvement. For example, the recent Demographic and Health Survey (DHS) data have shown 82% and 64% of the mothers fed exclusively to infants aged 0–6 months and 4–5 months, respectively [6]. However, the rate of EBF from rural Sri Lanka is still unclear.

Several studies reported that EBF by mothers from rural areas is unsatisfactory due to socio-economic status, maternal education, employment of mothers, mode of delivery of babies, birth weight of babies and early initiation of complementary feeding [7–10]. However, studies on breastfeeding practices, and the reasons for the discontinuation of desired period for breastfeeding are limited [11]. Though the health system in Jaffna District promotes awareness of breastfeeding to antenatal and postnatal mothers by Medical Officer of Health and Public Health Midwife levels of the Public Health Service, no studies on EBF or cessation of breastfeeding before six months in rural Jaffna had been reported. Indeed, the standard of living and health facilities of rural mothers of developing countries are low. Our previous study has shown that the prevalence of malnutrition was high in rural Jaffna [12]. The causes could be multifactorial, including the low rate of EBF. Thus, we hypothesize that lower numbers of rural mothers will feed breastmilk exclusively until the completion of six months and some factors will influence the lower rate. The findings of this study may help health authorities and policymakers of the Sri Lankan government to improve breastfeeding and its associated health status of rural children.

## Methods

### Study setting

A community-based descriptive cross-sectional study was conducted between 2013 and 2014 in Jaffna District, which is located in the Northern Province of Sri Lanka, 400 km away from the capital of Sri Lanka, Colombo. The Jaffna District is divided into 11 Medical Officer of Health (MOH) areas and is further divided into 84 Public Health Midwife areas in order to provide Public Health Services during the study period. The rural areas among these MOH areas were selected. The total population of Jaffna was 538,882, including 42,628 children under five years of age [13], and the population of the rural areas was 466,307. The ethnic majority of Jaffna speaks the Tamil language.

### Sample size and sampling technique

The sample size was calculated based on the equation z^2^p(1-p)/d^2^, where the Confidence Interval was set as 95%, the precision level as 5%, and the design effect as two [14]. A sample size of 340 was obtained after adding a 10% non-respondent rate.

Population-based multi-stage cluster sampling was used to identify the representative mothers with their children from Jaffna District. The primary sampling units were considered from all MOH areas. From a primary sampling unit, the required numbers of clusters (Public Health Inspector areas) were selected (secondary sampling unit) from the rural areas (*n* = 85). From a secondary sampling unit, the required number of households (*n* = 10) (tertiary sampling unit) [15] were selected using ‘spin a pen walk’ method [16]. In the absence of a child of less than 36 months of age, the next household which had children less than 36 months was selected. Eventually, the required number of mothers with their children (*n* = 340) were selected in proportion to the population from each of the selected Public Health Inspector areas.

In a house, if the mothers had more than one child under 36 months of age, the child with the most recent birthday was selected.

### Data collection and measures

The age, birth weight, and vaccination records were checked through the Child Health Development Record. Data on socio-economic and demographic factors; babies’ sex, birth weight, mode of delivery, and knowledge of the mothers on breastfeeding were obtained from all selected mothers using an interviewer-administered questionnaire. The interview was held at the mothers’ residences, and the questionnaire was administered in the Tamil language, as all mothers selected for this study in Jaffna District were Tamils at the time of data collection. Whenever the research team visited the selected mothers, they were informed before the visit. Under any unavoidable circumstances, if mothers informed the research team of their unavailability for visits due to unavoidable circumstances, and if mothers were not at home, the research team re-visited those houses.

**Fig. 1:**
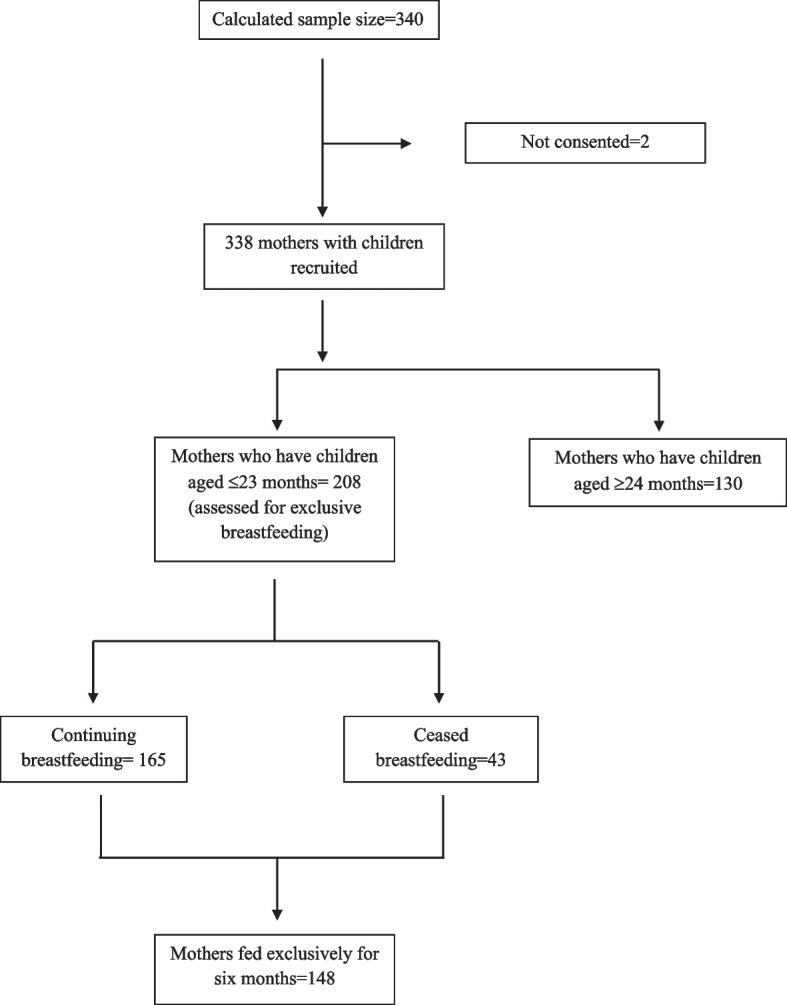
Flow diagram on recruitment of mothers to study the exclusive breastfeeding pattern

To assess the EBF rate and determinants of early cessation of breastfeeding exclusively and to minimize recall bias, mothers in a subgroup of households with babies aged less than 24 months (*n* = 208) were further asked the details of EBF and complementary feeding practices (Fig. 1). The EBF was determined as the children not receiving any food or drink, including complementary foods and formula milk or milk products, except for medicines and vitamins or mineral drops other than breast milk since birth [6]. If the mother breastfed exclusively for up to six months, it was recorded as ‘exclusively breastfed for six months’. Commencing complementary foods or infant formula before six months was considered as early cessation of EBF. However, we also recorded the breastfeeding duration to assess the extended breastfeeding for future analysis. To assess the prevalence of EBF, we adopted the ‘recall since birth’ method as it is widely used [17, 18]. As this method requires a lengthier recall period, the mothers were specifically questioned about the feeding patterns, including breastfeeding, to minimize recall bias. For instance, mothers were asked a series of questions, including: “How many months / years did you feed breast milk?”; “If yes, how many months did you feed your child only with breast milk, except medicines, vitamins or mineral drops, and formula milk or milk products?”; and “Did you give water or food or any other preparation to the child during the first six-months period and after?”, in order to obtain details on the EBF. The mothers were permitted to discuss how they fed their children, whereupon detailed follow-up questions were presented both to verify the validity of their claims and to support mothers’ recall of breastfeeding and complementary feeding.

In Sri Lanka, the third dose of oral poliovirus vaccine is administered at the age of six months. Hence, the mothers were questioned whether they exclusively breastfed their children by the time of the third dose of the vaccines. Details of complementary feeding practices, including solid and semi-solid foods, and initiation month of feeding were also obtained from mothers for cross-validation (data not shown). The data collectors initiated several measures, such as presenting show-cards of complementary foods to mothers and re-emphasizing the complementary feeding practices according to the guidelines of the Child Health Development Record booklet. In addition, the duration of extended breastfeeding was also asked from mothers who ceased EBF to cross-validate the results (data not shown).

Educational levels of the mothers were categorized into no formal education (no schooling), primary education (grades one to five), junior secondary education [grades six to General Certificate of Education (GCE)-Ordinary Level (O / L)], senior secondary [grades twelve to GCE- Advance Level (A / L)], senior secondary pass, and tertiary education (diploma, degree, and postgraduate degree).

### Statistical analysis

Descriptive statistics [such as mean (normal distribution), median (skewed distribution), standard deviation, and interquartile ratio (IQR)] were used. The Chi-square test at a 95% significance level was used to examine the association between the independent variables and the dependent variable of binary outcome (EBF up to six months or not). The odds ratio (OR) was calculated from the Chi-square test. The Multivariate logistic regression model to explore independent predictors of early cessation of EBF was used and the adjusted odds ratio (AOR) was calculated to evaluate the strength of the association. The significance of all analyses was assigned at *p* < 0.05. IBM-SPSS Statistics for Windows, Version 28.0 [Armonk, NY: IBM Corp.] package was used for statistical analysis.

## Results

### Information on the selected mothers and children

If the continuation of EBF was changed by providing complementary foods or products before six months, it was considered as cessation of EBF before six months. Even if the mother interrupted practicing EBF for a short while and then continued the EBF, this was also considered as cessation of EBF. The mean and median of the EBF duration were calculated.

The selected mothers (*n* = 338; two mothers did not agree to participate [response rate, 99.4%]) had 167 male children (49.4%), and the mean age of the total selected children was 24.2 (± 6.9) months. Low birth weight (LBW) (< 2500 g) babies were delivered by 16.3% (55 / 338) of the mothers. In this study, 88% gave birth vaginally. The majority of the mothers were Hindus (85.2%) (Table 1).

**Table 1:** Demographic details, and birthweight and mode of delivery of the mothers from the rural areas of Jaffna District who practiced EBF and the mean duration of EBF

Variable	Total (*n* = 338)		EBF (*n* = 208)								
	**No**	**%**	**No**^**#**^**. (Yes / Total)**	**%**^**¥**^	***p-*value**	**Mean Duration (Months)**	***p-*value**	**Crude OR (95% CI)**	***p-*value**	**AOR**	***p-*value**
***Sex***											
Male	167	49.4	69 / 102	67.6	0.288	5.13 (± 1.45)	0.390	0.72 (0.39–1.31)	0.288	-	-
Female	171	50.6	79 / 106	74.5		5.30 (± 1.47)		1			
***Type of family***											
Nuclear family	197	58.3	84 / 122	68.9	0.438	5.23 (1.38)	0.390	1.32 (0.71–2.44)	0.438	-	-
Extended family	141	41.7	64 / 86	74.4		5.20 (1.57)		1			
***Religion***											
Hindu	288	85.2	126 / 179	70.4	0.661	5.22 (1.44)	0.862	1.32 (0.53–3.28)	0.661	-	-
Christian	50	14.8	22 / 29	75.9		5.17 (1.58)		1			
***Birth weight***^***a***^											
NBW	283	83.7	134 / 176	76.1	0.000	5.39 (1.3)	0.000	1	0.000	1	0.006
LBW	55	16.3	14 / 32	43.8		4.28 (1.91)		4.1 (1.88–8.95)		3.70 (1.46–9.40)	
***Mode of delivery***											
Cesarean	41	12.1	16 / 27	51.6	0.017	4.48 (2.00)	0.002	2.75 (1.26–6.01)	0.017	3.04 (1.24–7.43)	0.015
Vaginal	297	87.9	132 / 181	74.6		5.34 (1.31)		1		1	
***Number of children***											
1	50	14.8	26 / 33	78.8	0.632	5.24 (± 1.75)	0.973	1	0.632	-	-
2	115	34.0	46 / 69	66.7		5.16 (± 1.39)		1.86(0.7–4.91)			
3	122	36.1	53 / 73	72.6		5.22 (± 1.5)		1.4(0.53–3.74)			
≤ 4	51	15.1	23 / 33	15.5		5.30 (± 1.46)		1.61(0.53–4.93)			

*EBF* exclusive breastfeeding, *LBW* low birth weight, *NBW* normal birth weight

^#^Number of mothers who fed breastfeeding exclusively

^**¥**^The percentage of exclusively breastfed children within the sex, type of family, religion, birth weight, mode of delivery, and number of children

^a^Birth weight; low birth weight (LBW) was defined as birth weight of a baby is less than 2500 g, NBW-normal birth weight ≥ 2500 g

The highest percentage of mothers had three children (36.1%), while 14.8% of mothers had only one child (Table 1). This study found that 8.0%, 85.5% and 5.9% of the mothers, respectively, had education up to primary, secondary and tertiary levels, while two mothers (0.6%) had never attended schools (Table 1).

The total family income included the total income of all family members. The median and mean monthly income were LKR (Sri Lankan Rupee) 20,000.00 [interquartile range (IQR) = 15,000.00] and LKR 24,351.04 (± 15,022.36), respectively.

Household assets were considered to construct wealth status based on the principal component analysis [19, 20] and classified as poor, second, middle, and fourth classes. The majority of the mothers (47.3%) were from the middle class and equal numbers were from the poor and fourth classes (11.5%) (Table 2).

**Table 2:** Demographic details and knowledge of EBF of mothers from the rural areas of Jaffna District and mean duration of EBF

Variable	Total (*n* = 338)		EBF (*n* = 208)								
	**No**	**%**	**No.**^**#**^** (No / Total)**	**%**^**¥**^	***p-*value**	**Mean EBF Duration (Months)**	***p-*value**	**Crude OR (95% CI)**	***p-*value**	**AOR**^**##**^	***p*-value**
***Education level of mothers ***^***a***^											
No formal / Primary	29	8.6	4 / 11	3.6	0.027	3.82 (± 1.51)	0.012	6.42 (1.62–25.62)	0.027	1.62 (0.26–9.93)	0.603
Junior Secondary	134	39.6	66 / 87	75.9		5.26 (± 1.51)		1.17 (0.52–2.61)		2.02 (0.32–12.76)	0.457
Senior Secondary	83	24.6	44 / 56	78.6		5.54 (± 1.03)		1		1	
Secondary Pass	72	21.3	26 / 40	65.0		5.03 (± 1.64)		1.97 (0.79–4.91)		0.52 (0.55–4.90)	0.568
Diploma / Degree	20	5.9	8 / 14	57.1		5.29 (± 1.0)		2.75 (0.8–9.47)		0.72 (0.14–3.58)	0.688
***Knowledge on EBF***^***b***^											
Yes	147	70.7	101 / 147	68.7	0.244	5.14 (1.54)	0.053	1	0.244	-	-
No	61	29.3	47 / 61	77.0		5.41 (1.24		1.53 (0.77–3.05)			
***Employment of mothers***^***c***^											
Unemployed	278	82.2	131 / 179	74.4	0.019	5.26 (± 1.47)	0.547	1	0.019	1	0.14
Professional	41	12.1	13 / 26	50.0		4.92 (± 1.44)		2.57 (1.9–5.56)		4.31 (1.34–13.9)	
Non-professional	19	5.6	4 / 6	66.7		5.33 (± 1.21)					
***Total income (LKR / Month)***^***d***^											
≤ 19,999	159	47.0	55 / 90	61.1	0.003	4.92 (± 1.6)	0.023	1	0.003	1	
20,000–39,999	131	38.8	78 / 94	83.0		5.51 (± 1.27)		0.32 (0.16–0.64)		0.34 (0.084–1.41)	0.139
≥ 40,000	48	14.2	15 / 24	62.5		5.17 (± 1.46)		0.94 (0.37–2.39)		1.20 (0.317–4.45)	0.799
***Wealth status***^***e***^											
Poor class	39	11.5	21 / 30	70.0	0.013	5.03 (± 1.67)	0.033	1	0.013	1	
Second class	100	29.6	48 / 65	73.8		5.34 (± 1.29)		0.83 (0.32–2.15)		2.33 (0.63–8.64)	0.206
Middle class	160	47.3	68 / 88	77.3		5.40 (± 1.36)		0.69 (0.27–1.73)		3.18(1.02–9.89)	0.046
Fourth class	39	11.5	11 / 25	44.0		4.48 (± 1.76)		2.97 (0.98–9.02)		2.95 (1.0–8.71)	0.051

*AOR* Adjusted odds ratio, *EBF* exclusive breastfeeding, *LKR* Sri Lankan rupee

^#^Number of mothers who fed breastfeeding exclusively

^**¥**^ The percentage of exclusively breastfed children within educational level of the mothers, knowledge on EBF, employment of mothers, total income and wealth status

^a^Primary level: grade 1–5; Junior secondary / up to general common examination-ordinary level (GCE [O / L]): from grade 6–11; Senior secondary / up to general common examination-advance level (GCE [A / L]): Grade 11–13; Diploma / degree holders: undergraduate or graduate degree or diploma holders

^b^Knowledge on EBF was collected from the mothers who have children aged between 6 to 23 months (*n* = 208)

^c^Professional job: skill work at government and non-government sectors, and mothers get regular monthly salary; Non-professional job: unskilled works, including non-skilled self-employments, and mothers get mostly daily wage

^d^Income is based on the Sri Lankan rupees (LKR)

^e^Household assets were used to obtain the wealth index based on principal component analysis (PCA) [20]

^##^Model summary for both Tables 1 and 2 for AOR; Model chi-square = 2.888 (*p-*value = 0.717), Nagelkerke R^2^ of the fitted model = 0.222

### Prevalence of exclusive breastfeeding for six months

Of the total of 338 mothers, only 208 mothers who had children between six and 23 months of age were selected for the assessment of EBF, as the other 130 mothers had children older than 24 months and found it difficult to recall the information on EBF (Fig. 1). EBF as recommended by the WHO was practiced by 71.2% of the mothers (180 days).

EBF was higher for female babies (53.4%) than for male babies (46.6%) (*p* ≥ 0.05) (Table 3). In this present study, 67.6% of male and 74.5% of female babies were exclusively breastfed (*p* ≥ 0.05). The mean birth weight of the EBF babies was 2947.48 (± 467.46) g, and 9.5% of the mothers had LBW babies (< 2500 g) (Table 3).

**Table 3:** Prevalence of EBF (*n* = 148) to the babies classified based on gender and birth weight, born by different mode of delivery to the mothers having different religions, type of families and number of children

EBF Period (Months)	Gender (No., %)				Birth weight^a^ (No., %)		Mode of delivery (No., %)		Type of family (No., %)			Religion (No., %)		No. of Children (No., %)				
	**Male**		**Female**		**NBW**	**LBW**	**Caesarian**	**Vaginal**	**Nuclear**		**Extended**	**Hindu**	**Christian**	**1**		**2**	**3**	** > 3**
A	69 (67.6)		79 (74.5)		134 (76.1)	14 (43.8)	16 (51.6)	132 (74.6)	84 (68.9)		64 (74.4)	126 (70.4)	22 (75.9)	26 (78.8)		46 (66.7)	53 (72.6)	23 (69.7)
B	46.6		53.4		90.5	9.5	10.8	89.2	56.8		43.2	85.1	14.9	17.6		31.1	35.8	15.5
**EBF period (months)**	**Educational Level (No., %)**						**Knowledge on EBF (No., %)**		**Employment**			**Monthly ncome (× 10,000) (No., %)**			**Wealth Index (No., %)**			
	**No Formal Educ**	**Primary**	**Junior Secondary**	**Senior Secondary**	**Secondary pass**	**Diploma or Degree**	**Yes**	**No**	**Unemployed**	**Professional**	**Non-Professional**	** ≤ 19.999**	**2.0–3.9999**	** ≥ 4.0**	**Poor**	**2**^**nd**^	**Middle**	**4th**
A	0 (0.0)	4 (44.4)	66 (75.9)	44 (78.6)	26 (65.0)	8 (57.1)	101 (68.7)	47 (77.0)	131 (74.4)	13 (50.0)	4 (66.7)	55 (61.1)	78 (83.0)	15 (62.5)	21 (70.0)	48 (73.8)	68 (77.3)	11 (44.4)
B	0.0	2.7	44.6	29.7	52.7	5.4	68.2	31.8	88.5	15.5	2.7	37.2	52.7	10.1	14.3	32.4	45.9	7.4

*EBF* exclusive breastfeeding, *LBW* low birth weight, *NBW* normal birth weight

A The numbers with percentage given in the raw based on the total respective sub-population (*n* = 208) analyzed for EBF; B Within exclusively breastfed population (%) (*n* = 148)

^a^Birth weight; low birth weight (LBW) was defined as birth weight of a baby is less than 2500 g, NBW normal birth weight ≥ 2500 g

Exclusive breastfeeding was practiced by 89.2% of mothers who had a vaginal delivery, while 10.8% of the mothers had a Caesarean section. Only 51.6% of the mothers who had Cesarean section exclusively breastfed.

Exclusive breastfeeding was practiced by 43.2% and 56.8% of mothers from extended and nuclear families, respectively (*p* ≥ 0.05) (Table 3). Of these mothers from 12.5% and 9.5%, respectively, had caesarean deliveries res.

In the selected population, 85.1% of mothers were Hindus. Exclusive breastfeeding was practiced by almost an equal percentage of Hindu (70.4%) and Christian (75.9%) mothers. However, among the exclusively breastfeeding group, were more Hindus (85.1%) than Christian mothers (14.9%) (Table 3).

Exclusive breastfeeding was practiced by more mothers who had one child (52%) than those with two or more children (Table 3). However, among the EBF mothers, those with three children were more in numbers (Table 3).

More of the mothers who had an education up to the senior secondary or higher level practiced EBF (50.3%) (Table 3). However, the mothers who had an educational level up to the junior secondary level were the highest among those who practiced EBF (44.6%), whereas 35% was the highest among those who had not EBF (*p* < 0.05).

Only 68.2% of the mothers who were aware of the WHO recommendation for the duration of EBF as six months practiced EBF (Table 3).

Among mothers, 47.1% of those who were unemployed and 50.0% who had professional jobs practiced EBF (Table 3). Of the employed and unemployed mothers who practiced EBF, 82.4% and 66.4%, respectively, had knowledge on EBF. The present study observed that 76.5% and 23.5% of the employed Hindus and Christian mothers, respectively and 86.3% and 13.7% of the unemployed Hindus and Christian mothers, respectively practiced EBF.

The majority of the 338 mothers were from families (47.0%) which had a monthly income of LKR < 19,999. Of the mothers who practiced EBF (*n* = 148), 37.2%, 52.7% and 10.1% were from families that had a monthly income of LKR 19,999.99, 20,000.00–39,999.99, and > 40,0000.00, respectively (Table 3).

The highest number of the mothers belonged to the middle class (*n* = 160), while a higher number of poor-classed mothers practiced EBF (53.8%). However, among those who practiced EBF, were more middle-classed mothers who practiced EBF followed by those from the second class. EBF was practiced by 70.0%, 73.8%, 77.3% and 44.0% of mothers from poor, second, middle, and fourth classes of wealth status, respectively (Table 3).

### Cessation of exclusive breastfeeding before six months (*n* = 60)

Results for mothers (*n* = 60) who did not abide by the WHO recommendation, to practice EBF for six months (28.8%) are shown in Tables 4 and 5. Table 4 gives information on the early cessation of EBF by 1^st^, 2^nd^, 3^rd^, 4^th^ and 5^th^ months. About 10% of the mothers ceased EBF by the 1^st^ month, while 28.3% of the mothers ceased EBF by the 3^rd^ month.

**Table 4:** Early cessation of EBF before completion of six months (*n* = 60), to the babies of different sex, birth weights and delivered by different modes by the mothers belonging to different types of families, religions, and having different number of children

	**EBF Period (months)**														
	**1**			**2**			**3**			**4**			**5**		
		**A**	**B**		**A**	**B**		**A**	**B**		**A**	**B**		**A**	**B**
***Sex***	**No**	**%**	**%**	**No**	**%**	**%**	**No**	**%**	**%**	**No**	**%**	**%**	**No**	**%**	**%**
Male	2	2.0	3.3	5	4.9	8.3	10	9.8	16.7	11	10.8	18.3	5	4.9	8.3
Female	4	3.8	8.3	4	3.8	6.7	7	6.6	11.7	2	1.9	3.3	10	9.4	16.7
***Birth weight***^***a***^															
LBW	3	1.7	5.0	6	3.4	10.0	13	7.4	21.7	7	4.0	11.7	13	7.4	21.7
NBW	3	9.4	5.0	3	9.4	5.0	4	12.5	8.3	6	18.8	10	2	6.3	3.3
***Mode of delivery***															
Cesarean	3	9.7	5.0	2	6.5	3.3	4	12.9	6.7	3	9.7	5	3	9.7	5.0
Vaginal	3	1.7	5.0	7	4.0	1.7	13	7.3	21.7	10	5.6	16.7	12	6.8	20.0
***Type of family***															
Nuclear	2	1.6	3.3	7	5.7	11.7	8	6.6	13.3	9	7.4	15	12	9.8	20
Extended	4	4.7	6.7	2	2.3	3.3	9	10.5	15.0	4	4.7	6.7	3	3.5	5.0
***Religion***															
Hindu	5	2.8	8.3	9	5.0	15	11	6.1	18.3	13	7.3	21.7	15	8.4	25
Christian	1	0.8	1.7	0	0.0	0.0	6	20.7	10.0	0	0.0	0.0	0	0.0	0.0
***No. of children***															
1	3	9.1	5.0	0	0.0	0.0	2	6.1	3.3	0	0.0	0.0	2	6.1	3.3
2	1	1.4	5.0	3	4.3	3.3	7	10.1	11.7	7	10.1	11.7	5	7.2	8.3
3	2	2.7	3.3	4	5.5	6.7	5	6.8	8.3	5	6.8	8.3	4	5.5	6.7
> 3	0	0.0	0.0	2	6.1	3.3	3	9.1	5.0	1	3.0	5	4	12.1	6.7
**Total**	**6**	**2.9**	**10.0**	**9**	**8.3**	**9**	**17**	**8.2**	**28.3**	**13**	**6.3**	**21.7**	**15**	**7.2**	**25**

*EBF* exclusive breastfeeding, *LBW* low birth weight, *NBW* normal birth weight

A The number and percentage given in the raw data based on the total respective sub-population (*n* = 208) analyzed for early cessation of EBF; B The percentage given in the raw based on the non-exclusively breastfed population (*n* = 60) for six months

^a^Birth weight; low birth weight (LBW) was defined as birth weight of a baby is less than 2500 g, NBW normal birth weight ≥ 2500 g

**Table 5:** Early cessation of EBF (*n* = 60) by the mothers having different educational levels, knowledge on EBF, employments, monthly total family income and wealth index

	**EBF Period (months)**														
	**1**			**2**			**3**			**4**			**5**		
		**A**	**B**		**A**	**B**		**A**	**B**		**A**	**B**		**A**	**B**
***Educational level***^***a***^	**No**	**%**	**%**	**No**	**%**	**%**	**No**	**%**	**%**	**No**	**%**	**%**	**No**	**%**	**%**
No formal	0	0.0	0.0	1	50.0	1.7	0	0.0	0.0	1	50.0	1.7	0	0.0	0.0
Primary	1	11.1	1.7	1	11.1	1.7	2	22.2	3.3	1	11.1	1.7	0	0.0	0.0
Junior	3	3.4	6.7	5	5.7	8.3	5	5.7	8.3	4	4.6	6.7	4	4.6	6.7
Senior secondary	0	0.0	0.0	1	1.8	1.7	5	8.9	1.7	1	1.8	1.7	5	8.9	8.3
Secondary pass	2	5.0	3.3	1	2.5	1.7	4	10.0	6.7	4	10.0	6.7	3	7.5	5.0
Diploma / degree	0	0.0	0.0	0	0.0	0.0	1	7.1	1.7	2	14.3	3.3	3	21.4	5.0
***Knowledge on EBF***^***b***^															
Yes	6	4.1	10	4	2.7	6.7	15	10.2	25.0	10	6.8	16.7	11	7.5	18.3
No	0	0.0	0.0	5	8.2	8.3	2	3.3	3.3	3	4.9	5.0	4	6.6	6.7
***Employment of mothers***^***c***^															
Unemployment	5	2.9	8.3	9	5.1	15.0	14	8.0	23.3	7	4.0	11.7	10	5.7	16.7
Professional	1	3.8	1.7	0	0.0	0.0	2	7.7	3.3	6	23.1	10.0	4	15.4	6.7
Non-professional	0	0.0	0.0	0	0.0	0.0	1	16.7	1.7	0	0	0	1	16.7	1.7
***Monthly income***^***d***^															
≤ 19,999	3	3.3	5.0	6	6.7	1.0	11	12.2	18.3	8	8.9	13.3	7	7.8	11.6
20, 000–39,999	2	2.1	3.3	3	3.2	5.0	4	4.3	6.6	3	3.2	5.0	4	4.3	6.6
≥ 40,000	1	4.2	1.6	0	0.0	0.0	0	0.0	0.0	2	8.3	3.3	2	8.3	3.3
***Wealth index***^***e***^															
Poor class	1	3.3	1.6	2	6.7	3.3	4	13.3	6.6	1	3.3	1.7	1	3.3	1.6
Second class	1	1.5	1.6	2	3.1	3.3	4	6.2	6.6	7	10.8	11.7	3	4.6	5.0
Middle class	2	2.3	3.3	4	4.5	6.6	5	5.7	8.3	1	1.1	1.7	8	9.1	13.3
Fourth class	2	8.0	3.3	1	4.0	1.6	5	16.6	8.3	4	16.0	6.7	3	12.0	5.0
**Total**	**6**	**2.9**	**10.0**	**9**	**8.3**	**9.0**	**17**	**8.2**	**28.3**	**13**	**6.3**	**21.7**	**15**	**7.2**	**25**

*EBF* exclusive breastfeeding, *LKR* Sri Lankan rupee, *PCA* principal component analysis

A The number and percentage given in the raw data based on the total respective sub-population (*n* = 208) analyzed for early cessation of EBF; B The percentage given in the raw based on the non-exclusively breastfed population (*n* = 60) for six months

^a^Primary level: grade 1–5; Junior secondary / up to general common examination-ordinary level (GCE [O / L]): from grade 6–11; Senior secondary / up to general common examination-advance level (GCE [A / L]): Grade 11–13; Diploma / degree holders: undergraduate or graduate degree or diploma holders

^b^Knowledge on EBF was collected from the mothers who have children aged between 6 to 23 months (*n* = 208). ^c^Professional job: skill work at government and non-government sectors, and mothers get regular monthly salary; Non-professional job: unskilled work, including non-skilled self-employment, and mothers get mostly daily wage

^d^Income is based on the Sri Lankan rupees (LKR)

^e^Household assets were used to obtain the wealth index based on principal component analysis (PCA) [20]

More mothers with male babies ceased EBF before the 4^th^ month, but during the 4^th^ month, the majority of the mothers with female babies ceased EBF (Table 4). Among the 60 mothers, more mothers with male babies (55%) ceased EBF before the completion of six months than those who had female babies (45%).

The mean birth weight of the babies who were not EBF for six months was 2779.48 (± 475.81) g. Among these babies, 30% had LBW babies (< 2500 g) (Table 4). EBF up to six months was more for normal birth weight babies than for the LBW babies (Table 4).

The early cessation of EBF was by 75% of the mothers who had normal vaginal deliveries. Almost the same number of mothers ceased EBF by the 2^nd^, 3^rd^, 4^th^ and 5^th^ months, while a higher number of mothers who had vaginal delivery ceased EBF by the 3^rd^, 4^th^ and 5^th^ months (Table 4).

A higher number of mothers from nuclear families ceased EBF (63.3%) than those from the extended families (36.7%) (*p* ≥ 0.05). The cessation of EBF by the mothers from nuclear families increased from the 1^st^ to the 5^th^ months, while the numbers fluctuated among the mothers from extended families (Table 4).

Among the 60 mothers who ceased EBF, only one Christian mother ceased EBF at the increased with time by the Hindu mothers. Among the mothers who ceased EBF early, 88.3% and 11.7% were Hindu or Christian, respectively (*p* ≥ 0.05). The Hindu and Christian mothers who had a Cesarean section and ceased EBF early were 26.4% and 14.3%, respectively, from extended families and 64.2% and 57.1%, respectively, were from nuclear families.

More mothers who had two (38.3%) or three (33.3%) children ceased EBF earlier than those with only one child (11.7%) (Table 4). The mothers with four or more children or fewer than four children and who ceased EBF early had 30.0% and 30.0% LBW children, respectively. In this study, more mothers with senior secondary level education (53.3%), ceased EBF than mothers with other levels of education (Table 5).

Among the mothers who had ceased EBF early, 76.7% were aware of the WHO recommended duration of EBF for six months and, among them, 23.9% had LBW babies, 19.6% had a Cesarean section, and 60.9% were from nuclear families (Table 5).

Among the mothers who ceased EBF early, 75.0% were unemployed (Table 5). More employed mothers (80.0%) who ceased EBF early had knowledge of EBF than unemployed mothers (75.6%). Further, 86.7% and 13.3% of the employed Hindu and Christian mothers, respectively, and 88.9% and 11.1% of the unemployed Hindu and Christian mothers, respectively, ceased EBF early. Among these mothers, 13.3%, 40.0%, and 46.7% of the employed mothers, respectively, had > 3, 3, and 2 children and among the unemployed mothers, and 17.8%, 31.1% and 35.6%, and 15.6%, respectively, had > 3, 3, and 2 children and one child (Table 5).

Of the mothers who ceased EBF early, the majority (58.3%) had a monthly family income of LKR 19,999.99 (Table 5). The majority of mothers who had a LBW baby (34.3%) who ceased EBF had a total monthly income of LKR < 19,999.00 followed by those who had LKR 19,999.00–39,999.99 (31.3%). Mothers who had a vaginal delivery (75.0%) and ceased EBF early had a total monthly income of LKR < 19,999.99 and had 22.2% of LBW babies.

Early cessation of EBF was highest (33.3%) among those who were from middle-class wealth status (Table 5). The mothers from the poor class who ceased EBF early had 55.6% LBW babies, followed by those from the fourth (42.9%) and second classes (35.6%).

## Discussion

Exclusive breastfeeding up to the six months from birth and continuing breastfeeding for up to two years are essential to fulfill the nutritional and physiological requirements of children [21]. Breast milk is cheaper and provides all the essential nutrients for poor infants in rural areas. However, rural mothers in many countries perform improper breastfeeding practices with early cessation, which may cause short- and long-term health effects in children [22]. Still, no studies have been reported regarding breastfeeding practices and factors influencing the breastfeeding of mothers from rural areas in Jaffna District, Sri Lanka. To our knowledge, this is the first study which explored EBF and early cessation of breastfeeding among the selected mothers. The present study aimed to assess the prevalence of EBF up to six months, the factors influencing EBF for six months, and the early cessation of breastfeeding before six months.

This study shows that the prevalence of EBF up to six months among the rural mothers was 71.2% based on “recall since birth”. It was previously reported that the prevalence of EBF until six months was 64.4% in Jaffna District [12]. The demographic and health survey (DHS) of Sri Lanka reported that the prevalence of EBF was 82%, but it was difficult to relate the DHS survey results with this study as DHS had obtained the data by a “24-h recall”, which can easily be an overestimation [18, 23]. The WHO reports that nearly 40.0% of infants up to six months are exclusively breastfed globally [4]. In the Kandy District of the Central Province of Sri Lanka, including rural and urban sectors, 50.8% of EBF practice was reported [6]. Other studies in Sri Lanka reported that the prevalence of EBF up to six months was 77.7% in the Naula MOH area, which is one of the MOH areas out of 13 in Matale District [24]; 71.3% in Gampaha District [25], 72.0% in the Kaduwela MOH area (1 / 18 MOH areas) in Colombo District [26], and 62.2% in the Ragama MOH area (1 / 16 MOH areas) in Gampaha District [27]. There are limited studies on EBF in rural Sri Lanka. The prevalence of EBF was 32.4% in rural plantation areas of Sri Lanka [28]. EBF of children in rural areas of other countries showed that, in India, the practice of EBF was 60.0% in rural Bangalore and Karnataka [29]; in a rural region of the Mysore District, Karnataka it was 48.5% [30] and in rural communities of central Gujarat it was 49.7% [31]. EBF for six months was practiced by 9.7% of mothers from a rural area of Al Der village in Kaliubia Governorate, Egypt [32]. It was observed that even though Jaffna District was affected by three consecutive decades of war, which ended in 2009 [33], the prevalence of EBF was higher in rural areas of Jaffna District when compared with the rural areas of other countries, while it was better or equal to other parts of Sri Lanka.

The gender of the babies had no influence on EBF by the mothers from rural areas in Jaffna District, which was consistent with other studies [34–36]. This indicated that the mothers from the rural areas in Jaffna District treat their male and female babies alike. There were contrasting reports to show that more male babies were exclusively breastfed in Ethiopia [37] and more female babies were exclusively breastfed in Nigeria [38]. A meta-analysis of studies in Ethiopia estimated that mothers of male babies had a 31.0% significantly higher chance of EBF during the first six months than mothers of female babies [39]. Thus, the variations in giving priority to the gender of the babies for EBF seem to be influenced by the myths and beliefs of different communities.

Among the LBW babies, 43.8% of the babies were exclusively breastfed. EBF up to six months was lower for the LBW babies than for the NBW babies. This could be due to the early initiation of formula milk to catch up growth [40]. It was reported that higher birth weight babies had a longer duration of EBF when compared with the lower birth weight babies [40]. Thus, the study indicated that the birth weight of the babies had an influence on the EBF or early cessation of breastfeeding.

The majority of the mothers who had a vaginal delivery practiced EBF (89.2%), while only 39.0% of the mothers who had a Cesarean section practiced EBF. The prevalence of EBF up to six months was lower among the mothers who had a Cesarean section than those who had a vaginal delivery. Higher number of mothers who had a Cesarean section ceased EBF before the end of six months as well as stopped breastfeeding before two years when compared with those who had a vaginal delivery [41]. Other studies have also reported a shorter duration of breastfeeding among the mothers who had a Cesarean section [42–44]. In Sri Lanka, the decision to opt for a Cesarean section has become a customary practice by mothers to select an auspicious time and date to deliver the baby.

EBF was practiced by 43.2% and 56.8% of mothers from extended and nuclear families, respectively (*p* ≥ 0.05). The present study showed that the early cessation of EBF was not associated with the type of family (nuclear or extended). It could be due to the living style of families in rural areas. For example, the interaction between adjacent households is higher in rural areas than in the urban areas of Jaffna. In rural areas, relatives live closer as people who live in colonies. Though the mothers are from nuclear families, their breastfeeding habits could have been influenced by the neighborhoods and by their relatives. Thus, living in nuclear or extended families is not much of a difference in the rural society of Jaffna District. In contrast, Velusamy et al. mentioned that mothers from extended families ceased EBF early compared to mothers from nuclear families [45].

In this study, it was observed that the prevalence of EBF was not influenced by the religion or of the mothers (43.8% Hindu and 44.0% Christian mothers). The present study found that the prevalence of EBF was slightly higher among Christian mothers (75.9%) than Hindu mothers (70.4%), and it was evident that religious practice may impact EBF practice in Jaffna. The findings concur with a study conducted in the USA that the religion of the mothers influenced the feeding practices in the USA [46]. For instance, babies of Catholic women had more risk of feeding formula preparation [46]. It has been reported that the cultural and religious practices among the mothers had an effect on the rate of EBF [47].

EBF was practiced by more mothers who had one child (52.0%) than those with two or more children. This clearly shows that the prevalence of EBF was high among mothers from small-sized families, indicating that they give more care to the first baby / single baby [48]. It was reported that the mothers who had two or more children ceased EBF early when compared with the mothers who had one child (adjusted hazard ratio = 1.26; *p* = 0.0001 [45]).

In this study, it was observed that most of the mothers on the senior secondary educational level exclusively breastfed for up to six months. It was coherent with other studies performed elsewhere [6, 49, 50]. Moreover, mothers who did not attend school did exclusively breastfed only for up to three months. It could be due to the unawareness of the importance of EBF. In this study, 2.7%, 53.3%, and 35.0% of the mothers who had the education to primary level or / and no formal education, up to the senior secondary, and junior secondary, respectively, ceased EBF early. The prevalence of EBF was 57.1% among the mothers who had an education up to tertiary level. This could be because 85.7% of mothers who had a higher educational level also have employment and had to return to work early [25, 42]. Thus, the mothers’ educational levels did influence the early cessation of EBF. Conversely, the educational level of the mothers from rural Ghana [48], and from Gampaha District, Sri Lanka [25] did not influence the EBF for up to six months.

This study found that 29.3% of the mothers were unaware that the duration of EBF recommended by the WHO is up to six months, as well as of the definition of EBF. Interestingly, the knowledge on EBF was not dependent on the educational levels of the mothers. It could be due to the instructions given by midwives from the respective MOH areas or the regular attendance of the mothers at the antenatal clinics. Among the mothers who knew the recommended duration of EBF as being six months, only 68.7% of the mothers exclusively breastfed. Though the mothers with the knowledge of EBF were more likely to EBF up to six months [51, 52], the association between the knowledge on EBF and prevalence of EBF was not significant (OR 1.53; 95% CI: 0.77, 3.05). Similar findings were observed elsewhere [6, 53, 54]. In contrast, all mothers from Kaduwela MOH area, a sub-urban area of Colombo District, were aware of the recommended duration of EBF [26].

The present study showed that employment of mothers from rural areas significantly influenced the early cessation of EBF. Working mothers of rural Jaffna had nearly a four-fold risk of discontinuation of EBF early compared to mothers who were not employed [6, 25, 27]. This could be due to the difference in maternity leave period provisions in government and non-government sectors. An employee in the government sector of Sri Lanka could receive 12 weeks (84 working days) of maternity leave benefits [55], while employees in the private sectors allow shorter maternity leave depending on the regulations of the institutions. It is also interesting to note that among the mothers who ceased EBF early, 75.0% were unemployed.

Of the mothers who ceased EBF early, the majority (58.3%) had a low monthly family income. The majority of the LBW babies were delivered by the low-income mothers (34.3%) who ceased EBF. Our findings were coherent with others finding [56, 57].

This study observed that mothers with higher wealth status practiced the early introduction of infant formula milk (56.0%) before six months from birth, and 44.0% of the mothers from the fourth wealth class exclusively breastfed, while 77.3% of the mothers from the middle class exclusively breastfed. Mothers from a higher wealth class could afford to buy infant formula, which is generally expensive in Jaffna [12, 58]. A study on upper-class Mexican mothers revealed that they discontinued EBF at 3.3 (± 2.1) months [51].

There were some limitations. The assessment of the duration of EBF in the present study was based on “recall since birth” method [18]. Kelkay et al. [59] stated that “the last 24-h dietary practice preceding the interview” is a better method to assess the prevalence of EBF. However, Fenta et al. [18] reported that seven days of the “24-h recall” method is close to “recall since birth” since a single “24-h recall” overestimates the prevalence of EBF. Abdel-Hady et al. [17] claimed that both “recall since birth” and “24-h recall” methods were required to be reported to assess the full picture of breastfeeding patterns. However, as there are still no definite methods to assess the prevalence of EBF, the recall method has been followed. To assess the prevalence of EBF, we selected mothers who have children aged 6–23 months from the total population to avoid recall bias. The prospective cohort design may be a better method to describe the prevalence of EBF. We were unable to carry out the cohort study due to financial constraints and time restrictions. However, the follow-up study may motivate the mothers to practice EBF for longer periods of extended breastfeeding. We also did not select the mothers who have babies less than six months as our study focused on the prevalence of EBF up to six months as per WHO recommendation. Thus, we selected the babies who had completed six months of age or more. Our study might have some recall bias as some mothers had to recall more than a year ago. However, our research team considered several precautionary measures to reduce the recall bias as described in the methodology. The maternal age and marital status would have had some impact on the early cessation of breastfeeding. Since our study design was a cross-sectional design, the cause and effect cannot be inferred from the findings.

## Conclusion

The present study revealed that the prevalence of EBF up to six months (71.2%) was observed to be low in rural babies of Jaffna District when compared with the National prevalence. Factors, such as lower maternal education level, maternal employment, babies born as LBW, child delivered by Cesarean section, and high-income households influenced the early cessation of EBF. Findings from the present study would assist the Regional Directorate of Health Service of Jaffna with policymakers in increasing maternity leave to at least six months; reducing the Cesarean section rate; providing nutritional support to pregnant mothers and educating mothers of rural Jaffna on proper breastfeeding and the right initiation of complementary feeding.

## Data Availability

The datasets used and / or analyzed during the current study are available from the corresponding author on reasonable request.

## Abbreviations

EBF: Exclusive breastfeeding
LBW: Low birth weight
NBW: Normal birth weight
LKR: Sri Lankan rupee
MOH: Medical Officer of Health
WHO: World Health Organization

## References

[1] Martin CR, Ling P-R, Blackburn GL (2016). Review of infant feeding: key features of breast milk and infant formula. Nutrients.

[2] Elyas L, Mekasha A, Admasie A, Assefa E (2017). Exclusive breastfeeding practice and associated factors among mothers attending private pediatric and child clinics, Addis Ababa, Ethiopia: a cross-sectional study. Int J Pediatr.

[3] Palmeira P, Carneiro-Sampaio M (2016). Immunology of breast milk. Rev Assoc Med Bras.

[4] World Health Organization. Breastfeeding 2022. Available from: https://www.who.int/health-topics/breastfeeding#tab=tab_1. Accessed 11 June 2023.

[5] The World Breastfeeding Trends Initiative (WBTi). Press Briefing: The World Breastfeeding Trends Initiative (WBTi), Congratulates Sri Lanka on achieving first “Green” nation status supporting breastfeeding women. 2020. p. 4–6. https://www.worldbreastfeedingtrends.org/uploads/resources/document/wbti-press-release-9-jan-2020.pdf. Accessed 11 June 2023.

[6] Ratnayake HE, Rowel D (2018). Prevalence of exclusive breastfeeding and barriers for its continuation up to six months in Kandy district. Sri Lanka Int Breastfeed J.

[7] Babaee E, Eshrati B, Asadi-Aliabadi M, Purabdollah M, Nojomi M (2020). Early cessation of breastfeeding and determinants: time to event analysis. J Nutr Metab.

[8] Kabir A, Maitrot MRL (2017). Factors influencing feeding practices of extreme poor infants and young children in families of working mothers in Dhaka slums: a qualitative study. PLoS ONE.

[9] Noh J-W, Kim Y, Akram N, Yoo K-B, Cheon J, Lee LJ (2019). Factors affecting breastfeeding practices in Sindh province, Pakistan: a secondary analysis of cross-sectional survey data. Int J Environ Res Public Health.

[10] Yılmaz E, Öcal FD, Vural Yılmaz Z, Ceyhan M, Kara OF, Küçüközkan T (2017). Early initiation and exclusive breastfeeding: factors Influencing the attitudes of mothers who gave birth in a baby friendly Hospital. Turk J Obstet Gynecol.

[11] Odom EC, Li R, Scanlon KS, Perrine CG, Grummer-Strawn L (2013). Reasons for earlier than desired cessation of breastfeeding. Pediatrics.

[12] Karthigesu K, Sandrasegarampillai B, Arasaratnam V (2017). Breastfeeding practices and nutritional status of children aged one to five years in Jaffna District. Sri Lanka Indian J Nutr Diet.

[13] Census of Population and Housing, Department of Census and Statistics, Ministry of Finance and Planning, Sri Lanka. http://www.statistics.gov.lk/PopHouSat/CPH2011/Pages/Activities/Reports/CPH_2012_5Per_Rpt.pdf. Accessed 11 June 2023.

[14] Lwanga SK, Lemeshow S, World Health Organization. Sample size determination in health studies: a practical manual. World Health Organization; 1991. https://apps.who.int/iris/handle/10665/40062. Accessed 11 June 2023.

[15] Karthigesu K, Sandrasegarampillai B, Arasaratnam V (2021). Factors influencing the iodine status of children aged 12 to 59 months from Jaffna District, Sri Lanka in the post-iodization era; a descriptive, cross-sectional study. PLoS ONE.

[16] Bostoen K, Chalabi Z (2006). Optimization of household survey sampling without sample frames. Int J Epidemiol.

[17] Abdel-Hady DM, El-Gilany A-H (2016). Calculating exclusive breastfeeding rates: comparing dietary “24-hour recall” with recall “since birth” methods. Breastfeeding Med.

[18] Fenta EH, Yirgu R, Shikur B, Gebreyesus SH (2017). A single 24 h recall overestimates exclusive breastfeeding practices among infants aged less than six months in rural Ethiopia. Int Breastfeed J.

[19] McKenzie DJ (2005). Measuring inequality with asset indicators. J Popul Econ.

[20] Vyas S, Kumaranayake L (2006). Constructing socio-economic status indices: how to use principal components analysis. Health Policy Plan.

[21] World Health Organization. Infant and young child feeding: model chapter for textbooks for medical students and allied health professionals. 2009. https://apps.who.int/iris/handle/10665/44117. Accessed 11 June 2023. 23905206

[22] Hailu WS, Bayih MT, Babble NF (2020). Four in every ten infants in Northwest Ethiopia exposed to sub-optimal breastfeeding practice. PLoS ONE.

[23] Agampodi SB, Agampodi TC, De Silva A (2009). Exclusive breastfeeding in Sri Lanka: problems of interpretation of reported rates. Int Breastfeed J.

[24] Agampodi SB, Fernando S, Dharmaratne SD, Agampodi TC (2011). Duration of exclusive breastfeeding; validity of retrospective assessment at nine months of age. BMC Pediatr.

[25] Perera PJ, Ranathunga N, Fernando MP, Sampath W, Samaranayake GB (2012). Actual exclusive breastfeeding rates and determinants among a cohort of children living in Gampaha district Sri Lanka: a prospective observational study. Int Breastfeed J.

[26] Dhammika BL, Gunawardena N (2012). Knowledge, practices and concerns regarding exclusive breastfeeding for six months among mothers of infants in a suburban setting in Sri Lanka. Sri Lanka J Child Healt.

[27] Perera PJ, Fernando M, Warnakulasuria T, Ranathunga N (2011). Feeding practices among children attending child welfare clinics in Ragama MOH area: a descriptive cross-sectional study. Int Breastfeed J.

[28] Sørensen E, Fernando DN, Hettiarachchi I, Durongdej S, Podhipak A, Skaara BB (1998). Exclusive breastfeeding among women on the plantations in Sri Lanka. J Trop Pediatr.

[29] Madhu K, Chowdary S, Masthi R (2009). Breast feeding practices and newborn care in rural areas: a descriptive cross-sectional study. Indian J Community Med.

[30] Nishimura H, Krupp K, Gowda S, Srinivas V, Arun A, Madhivanan P (2018). Determinants of exclusive breastfeeding in rural South India. Int Breastfeed J.

[31] Bhanderi DJ, Pandya YP, Sharma DB (2019). Barriers to exclusive breastfeeding in rural community of central Gujarat. India J Family Med Prim Care.

[32] Al Ghwass MME, Ahmed D (2011). Prevalence and predictors of 6-month exclusive breastfeeding in a rural area in Egypt. Breastfeed Med.

[33] Siriwardhana C, Wickramage K (2014). Conflict, forced displacement and health in Sri Lanka: a review of the research landscape. Confl Health.

[34] Gizaw Z, Woldu W, Bitew BD (2017). Exclusive breastfeeding status of children aged between 6 and 24 months in the nomadic population of Hadaleala district, Afar Region. Northeast Ethiopia Int Breastfeed J.

[35] Hitachi M, Honda S, Kaneko S, Kamiya Y (2019). Correlates of exclusive breastfeeding practices in rural and urban Niger: a community-based cross-sectional study. Int Breastfeed J.

[36] Stamenkovic Z, Matejic B, Djikanovic B, Bjegovic-Mikanovic V (2020). Surprising differences in the practice of exclusive breastfeeding in non-Roma and Roma population in Serbia. Front Public Health.

[37] Jama A, Gebreyesus H, Wubayehu T, Gebregyorgis T, Teweldemedhin M, Berhe T (2020). Exclusive breastfeeding for the first six months of life and its associated factors among children age 6–24 months in Burao district. Somaliland Int Breastfeed J.

[38] Agho KE, Dibley MJ, Odiase JI, Ogbonmwan SM (2011). Determinants of exclusive breastfeeding in Nigeria. BMC Pregnancy Childbirth.

[39] Habtewold TD, Sharew NT, Alemu SM (2019). Evidence on the effect of gender of newborn, antenatal care and postnatal care on breastfeeding practices in Ethiopia: a meta-analysis and meta-regression analysis of observational studies. BMJ Open.

[40] Flaherman VJ, McKean M, Cabana MD (2013). Higher birth weight improves rates of exclusive breastfeeding through 3 months. Infant Child Adolesc Nutr.

[41] Chen C, Yan Y, Gao X, Xiang S, He Q, Zeng G (2018). Influences of cesarean delivery on breastfeeding practices and duration: a prospective cohort study. J Hum Lact.

[42] Agampodi TC, Dharmasoma NK, Koralagedara IS, Dissanayaka T, Warnasekara J, Agampodi SB (2021). Barriers for early initiation and exclusive breastfeeding up to six months in predominantly rural Sri Lanka: a need to strengthen policy implementation. Int Breastfeed J.

[43] Paksoy Erbaydar N, Erbaydar T (2020). Relationship between caesarean section and breastfeeding: evidence from the 2013 Turkey demographic and health survey. BMC Pregnancy Childbirth [Internet].

[44] Qiu L, Binns C, Zhao Y, Lee A, Xie X (2008). Breastfeeding following caesarean section in Zhejiang Province: public health implications. Asia Pac J Public Health.

[45] Velusamy V, Premkumar PS, Kang G (2017). Exclusive breastfeeding practices among mothers in urban slum settlements: pooled analysis from three prospective birth cohort studies in South India. Int Breastfeed J.

[46] Bernard JY, Rifas-Shiman SL, Cohen E, Lioret S, de Lauzon-Guillain B, Charles M (2020). Maternal religion and breastfeeding intention and practice in the US Project Viva cohort. Birth.

[47] Agampodi SB, Agampodi TC, Piyaseeli UKD (2007). Breastfeeding practices in a public health field practice area in Sri Lanka: a survival analysis. Int Breastfeed J.

[48] Manyeh AK, Amu A, Akpakli DE, Williams JE, Gyapong M (2020). Estimating the rate and determinants of exclusive breastfeeding practices among rural mothers in Southern Ghana. Int Breastfeed J.

[49] Laksono AD, Wulandari RD, Ibad M, Kusrini I (2021). The effects of mother’s education on achieving exclusive breastfeeding in Indonesia. BMC Public Health.

[50] Wang L, Van Grieken A, Van Der Velde LA, Vlasblom E, Beltman M, L’Hoir MP (2019). Factors associated with early introduction of complementary feeding and consumption of non-recommended foods among Dutch infants: the BeeBOFT study. BMC Public Health.

[51] Ávila-Ortiz MN, Castro-Sánchez AE, Martínez-González EA, Núñez-Rocha GM, Zambrano-Moreno A (2020). Factors associated with abandoning exclusive breastfeeding in Mexican mothers at two private hospitals. Int Breastfeed J.

[52] Senghore T, Omotosho TA, Ceesay O, Williams DCH (2018). Predictors of exclusive breastfeeding knowledge and intention to or practice of exclusive breastfeeding among antenatal and postnatal women receiving routine care: a cross-sectional study. Int Breastfeed J.

[53] Mogre V, Dery M, Gaa PK (2016). Knowledge, attitudes and determinants of exclusive breastfeeding practice among Ghanaian rural lactating mothers. Int Breastfeed J.

[54] Rana MM, Islam MR, Karim MR, Islam AZ, Haque MA, Shahiduzzaman M (2020). Knowledge and practices of exclusive breastfeeding among mothers in rural areas of Rajshahi district in Bangladesh: a community clinic based study. PLoS ONE.

[55] Maternity Benefits (Amendment) Act, No. 15 Of 2018, Parliament of the Democratic Socialist Republic of Sri Lanka, published as a supplement to part II of the gazette of the Democratic Socialist Republic of Sri Lanka. http://eohfs.health.gov.lk/occupational/images/pdf/acts_regulations/maternity_benifit_ordinance.pdf. Accessed 11 June 2023.

[56] Shofiya D, Sumarmi S, Ahmed F (2020). Nutritional status, family income and early breastfeeding initiation as determinants to successful exclusive breastfeeding. J Public Health Res.

[57] Tewabe T, Mandesh A, Gualu T, Alem G, Mekuria G, Zeleke H (2016). Exclusive breastfeeding practice and associated factors among mothers in Motta town, East Gojjam zone, Amhara Regional State, Ethiopia, 2015: a cross-sectional study. Int Breastfeed J.

[58] Rothstein JD, Winch PJ, Pachas J, Cabrera LZ, Ochoa M, Gilman RH (2021). Vulnerable families and costly formula: a qualitative exploration of infant formula purchasing among peri-urban Peruvian households. Int Breastfeed J.

[59] Kelkay B, Kindalem E, Tagele A, Moges Y (2020). Cessation of exclusive breastfeeding and determining factors at the University of Gondar Comprehensive Specialized Hospital. Northwest Ethiopia Int J Pediatr.

